# STI Health Disparities: A Systematic Review and Meta-Analysis of the Effectiveness of Preventive Interventions in Educational Settings

**DOI:** 10.3390/ijerph15122819

**Published:** 2018-12-11

**Authors:** Nway Mon Kyaw Soe, Yelena Bird, Michael Schwandt, John Moraros

**Affiliations:** 1 School of Public Health, University of Saskatchewan, Saskatoon, SK S7N 2Z4, Canada; nwk504@mail.usask.ca (N.M.K.S.); yelena.bird@usask.ca (Y.B.); 2 Medical Health Officer, Fraser Health Authority, Surrey, BC V3T 0H1, Canada; michael.schwandt@fraserhealth.ca

**Keywords:** STIs, youth, educational settings, health disparities, preventive interventions, effectiveness

## Abstract

The purpose of this systematic review and meta-analysis was to address disparities related to sexual health among students by examining the effectiveness of sexually transmitted infection (STI) preventive interventions in educational settings. PubMed, Medline, Cochrane Library, Public Health Database, and EMBASE databases were used to conduct searches. Information relating to studies, programs, participants, and quantitative outcome variables were extracted. Risk of bias was assessed and meta-analysis was conducted. This systematic review included 16 articles. The outcomes were classified into behavioral and psychosocial categories. The behavioral category included sexual partners, sexual activity, condom use, STI/HIV testing, and alcohol/drug use before sex. The psychosocial category consisted of knowledge, motivational factors, and skills. Interventions had a significantly positive impact on both behavioral (OR, 1.28; 95% CI, 1.17–1.39) and psychosocial (OR, 1.92; 95% CI, 1.36–2.72) outcomes. Among the psychosocial outcomes, the interventions were most effective at promoting knowledge (OR, 3.17; 95% CI, 2.13–4.72), followed by enhancing motivational factors (OR, 1.69; 95% CI, 1.04–2.75) and increasing behavioral skills (OR, 1.43; 95% CI, 1.13–1.81). The results of this systematic review provide empirical evidence for public health professionals and policy makers regarding planning, implementation, evaluation, and modification of STI preventive intervention programs in educational settings.

## 1. Introduction

Sexually transmitted infections (STIs) are a significant public health concern worldwide [1]. According to the World Health Organization (WHO), more than 360 million people acquire one of four STIs (chlamydia, gonorrhea, syphilis, and trichomonas) annually [2]. Youth (aged 15–24 years old) are particularly vulnerable to STIs due to their high likelihood to engage in risky behaviors [3,4]. The majority of youth are students, especially in developed countries [5,6,7,8]. Therefore, educational institutions represent ideal settings to implement effective strategies to help reduce the STI burden and provide improved health to their students.

Youth in developed countries are not immune to the scourge of STIs. In the U.S., individuals 25 years old and younger accounted for half of all STI cases despite representing only a quarter of the sexually active population [9]. Similarly, in Canada, individuals aged 15–29 years old reported the highest rates among three commonly notifiable STIs (chlamydia, gonorrhea, and syphilis) [10]. In Australia, 77% of chlamydia incidence cases in 2015 were seen among individuals aged 15–29 years old and the highest rates of gonorrhea and syphilis were reported among males aged 20–29 years old [11]. In Europe, youth accounted for 62% of chlamydia and 52% of gonorrhea cases [12]. These disproportionately high STI rates suggest deficits related to sexual health among youth.

Inadequate knowledge, risky behaviors, and lack of access to sexual health programming and services contribute to the high rates of STIs observed among youth. According to the Canadian Youth, Sexual Health and HIV/AIDS Study (CYSHHAS), approximately half of grade nine students (14–16 years old) did not know that HIV has no cure, and STI risk perception had little influence on engaging in safe sexual practices [13]. A research study conducted among secondary students in Italy also found that only 65.3% and 46.6% of the respondents correctly recognized syphilis and herpes from a given list of STIs [14]. Similarly, 46% of German ninth graders had no knowledge about chlamydia [15]. Previous studies in the U.S. found that youth are not practicing consistent condom use and instead favour the use of birth control methods that prevent unwanted pregnancies but offer no protection against STIs [16,17,18]. These research findings are supported by statistics suggesting that 40% of sexually active high school students in the U.S. [19] and 33% of Canadian youth did not use a condom during their last sexual encounter [20]. In Italy and Germany, less than 40% of sexually active students used condoms consistently [14,15]. It is reported that 11.5% of U.S. high school students had four or more sexual partners in their lifetime and nearly 4% had early sexual initiation (before the age of 13 years old) [19]. Likewise, approximately one-third of Canadian youth reported having multiple sexual partners in the last 12 months and 9% had early sexual initiation (before the age of 15 years old) [20,21]. According to Italian data, 26% of high school students had two or more sexual partners in the last two years and nearly 50% had sexual initiation before the age of 15.5 years old [14]. 

It is widely acknowledged that in order to be successful, preventive efforts require behavioral change [16]. Currently, there are numerous biomedical and structural barriers affecting the prevention, diagnosis, and treatment of STIs. Biomedical barriers impacting STI interventions are due to the lack of technological advances in comprehensively addressing STIs (i.e., all-in-one screening tests, vaccines, and curative treatments) [1,16,22,23]. Structural barriers impacting STI interventions are due to health disparities and policies affecting accessibility and viability of services to youth (i.e., funding cutbacks, lack of infrastructure, ineffective messaging, and inefficient intervention strategies) [24,25,26]. Furthermore, many STIs are difficult to control once an individual is infected because of their asymptomatic nature, drug resistance, social stigmatization, and confidentiality issues [27,28,29]. 

To implement equitable and effective preventive interventions to reduce the risk of STIs among youth, educational institutions are recognized as ideal settings [9,30,31,32]. These settings provide the necessary social framework, accessibility, and educational opportunities for sexual health and promotion initiatives to specifically target youth [31,32]. However, in the U.S. fewer than half of the high schools and only one-fifth of the middle schools are reported to teach the essential sexual education topics (i.e., relationships, sexual abstinence, condom use, negotiation, pathology and transmission of HIV, and related information on other common STIs) [33] and provide access to sexual health services as recommended by the Centers for Disease Control and Prevention (CDC) [9]. Australia faces similar challenges, with reports of significant gaps in the current sexual health education programmes and a growing need to improve services among students [34,35]. In Canada, only a few high schools have well-established sexual health curricula [29] and the outcomes to date have been unsatisfactory [36,37,38]. In a recent Italian survey, 59% of secondary students reported that the sexual health education programs at their schools were either insufficient or non-existent [17]. 

STI preventive interventions are also needed at post-secondary institutions. In the U.S., post-secondary students show poor knowledge, low condom use, and a high tendency to engage in unsafe sexual practices [39,40,41,42,43,44]. Decreases in condom use were also seen among Canadian students, especially as they transitioned from high school to post-secondary institutions, where less than half reported using a condom during their last sexual encounter [31]. Therefore, to equitably and effectively reduce the burden of STIs, preventive interventions that increase knowledge and promote behavioral change including safe sex practices are considered the gold standard. By introducing STI preventive interventions to youth, it is likely they may engage in safe sex practices throughout their lifetime. The purpose of this systematic review and meta-analysis was to address disparities related to sexual health among students by examining the effectiveness of STI preventive interventions in educational settings.

## 2. Materials and Methods 

### 2.1. Search Strategy and Study Selection

A literature search was conducted using the following electronic databases: PubMed, Medline, Cochrane Library, Public Health Database, and EMBASE. The following keywords and PubMed MeSH terms were used: HIV, chlamydia, chlamydia infections, gonorrhea, syphilis, sexually transmitted diseases, mass screening, health promotion, health education, health disparity, guideline adherence, preventive health services, community health planning, health plan implementation, population characteristics/prevention and control, health education, health knowledge/attitudes and practice, program effectiveness, cost effectiveness, health impact assessment, cost savings, and evaluation studies as topics. 

Articles obtained from the systematic search were screened in two steps: (1) title and abstract screening and (2) full-text screening. Dual screening was employed, whereby two authors initially screened 20 articles to determine the consistent use of the inclusion and exclusion criteria. The two authors independently conducted title and abstract screening followed by full-text screening. Discrepancies in decisions between the screeners were initially discussed among themselves, and when consensus was not achieved, a tie-breaking vote was cast by the third author.

### 2.2. Inclusion and Exclusion Criteria

Articles were included if they satisfied the following criteria: publicly available; peer-reviewed; published online between 2007 and 2017; English language; human participants; educational settings; examining STIs or chlamydia or gonorrhea or syphilis or HIV; preventive interventions; quantitative outcome measurements; and data from North America, Europe, and Oceania. Articles involving case reports or case series were excluded.

### 2.3. Data Extraction

Information extracted from the selected articles included in our study were: authors, publication year, location, program types, type of providers, settings, type of study, number of participants, demographics (age, sex, ethnicity), and the quantitative data of the outcome variables, which assessed the effectiveness of the interventions. If there were more than one follow-up measurement, we extracted data only from the final follow-up. Data were collected into a common folder and shared between the researchers. Spreadsheets were constructed based on outcomes of interest and data extracted from the final articles.

### 2.4. Risk of Bias Assessment

Risk of bias was assessed independently by two of the authors by applying the specific criteria recommended by the Agency for Healthcare Research and Quality (AHRQ) [45]. The criteria were used to assess five types of bias: selection, performance, attrition, detection, and reporting.

### 2.5. Data Analysis

In our study, odds ratio (OR) was used as the principle effect size, with values >1 reflecting a positive effect of the STI preventive intervention on the outcomes of interest. Crude effect sizes were computed when adjusted ones were not available. Adjusted ORs were used to provide a conservative effect estimate and included age, gender, ethnicity, and parental education. 

Pooled estimates were obtained using random effects models to account for heterogeneity. Analysis of heterogeneity was conducted using *I*^2^ tests and Q-statistics to assess the degree of true variation of the effect size among studies [46,47]. Influential analysis was conducted to determine the robustness and effect that each individual study had on the overall pooled estimate. Pooled, comparative, and sub-group meta-analysis was conducted using the Comprehensive Meta-Analysis (CMA) software version 3 (Biostat Inc., Englewood, NJ, USA).

## 3. Results

### 3.1. Study Selection

**Figure 1:**
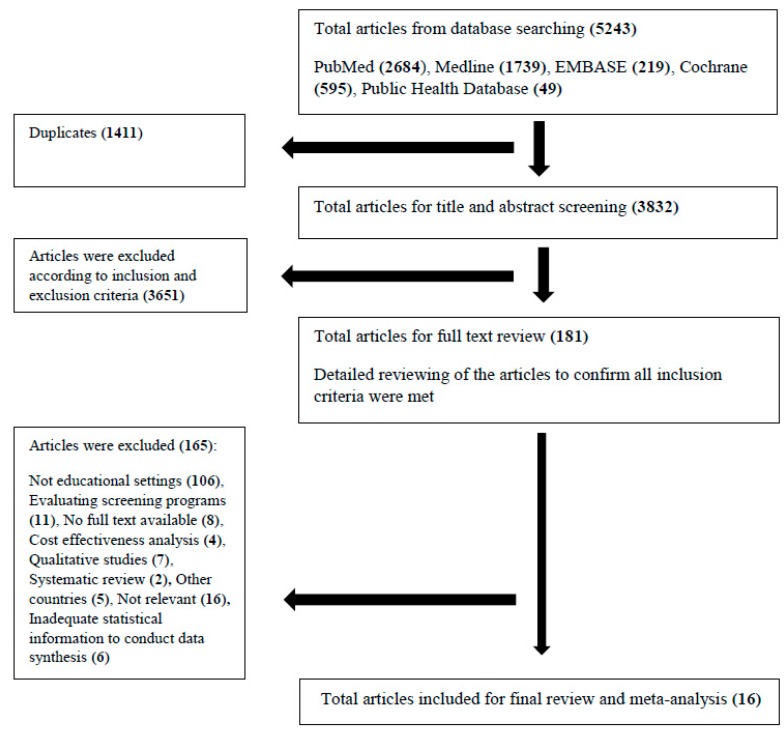
PRISMA flow diagram for included studies.

A total of 5243 articles were identified after an initial search of the electronic databases. Among those, 1411 articles were removed as duplicates. The remaining 3832 articles underwent title and abstract screening and upon completion, 181 articles qualified for full-text review. Guided by the inclusion and exclusion criteria determined a priori, 165 articles were further excluded. Finally, 16 articles were deemed appropriate and were selected for further analysis (Figure 1).

### 3.2. Risk of Bias Assessment

There were 16 articles selected, of which four studies were determined to have a low risk of bias [47,48,49,50,51], eight a moderate risk of bias [52,53,54,55,56,57,58,59], and four a high risk of bias [60,61,62,63]. The main methodological concerns were focused on performance bias (15 studies) [49,50,51,52,53,54,55,56,57,58,59,60,61,62,63] and detection bias (6 studies) [55,56,58,60,61,62] (Table 1).

### 3.3. Study Characteristics

In total, there were 16 studies included in our systematic review and meta-analysis, encompassing 15 different STI preventive programs (two studies overlapped by examining the same program [49,54]). The majority of the programs were in the U.S. and conducted in middle school (two), high school (six), and post-secondary (three) settings. The rest of the programs were conducted in other countries. Most programs were guided by health promotion theories and promoted both knowledge acquisition and improved behavioral skills among participating students. Two-thirds of the programs were conducted face-to-face and one-third were technology-based interventions. There was a peer-to-peer component in seven programs. The duration of the program interventions ranged from 1–18 h. Program interventions were evaluated at designated time interval(s): immediately, 3-months, 6-months, and 12-months. Table 2 provides a summary description of the included studies.

### 3.4. Synthesis of Results

All 16 included studies measured psychosocial outcomes; 10 studies also measured behavioral outcomes, but no studies measured biological outcomes. Synthesis of effect measures was conducted for behavioral outcomes (overall), psychosocial outcomes (overall) and its sub-categories (information/knowledge, motivational factors, and behavioral skills).

#### 3.4.1. Effects of Interventions on the Behavioral and Psychosocial Outcomes

**Figure 2:**
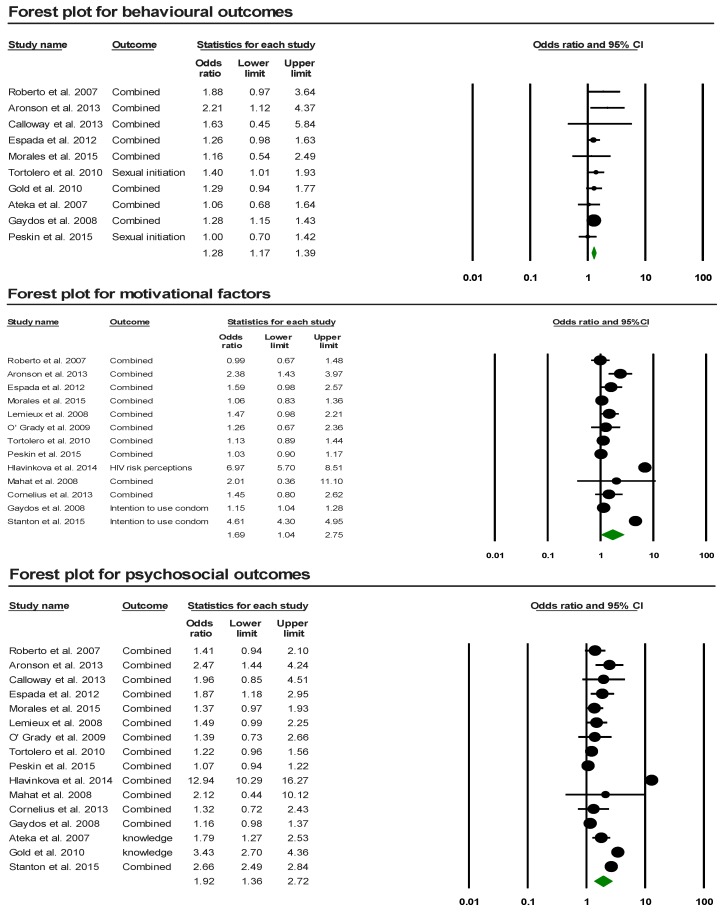

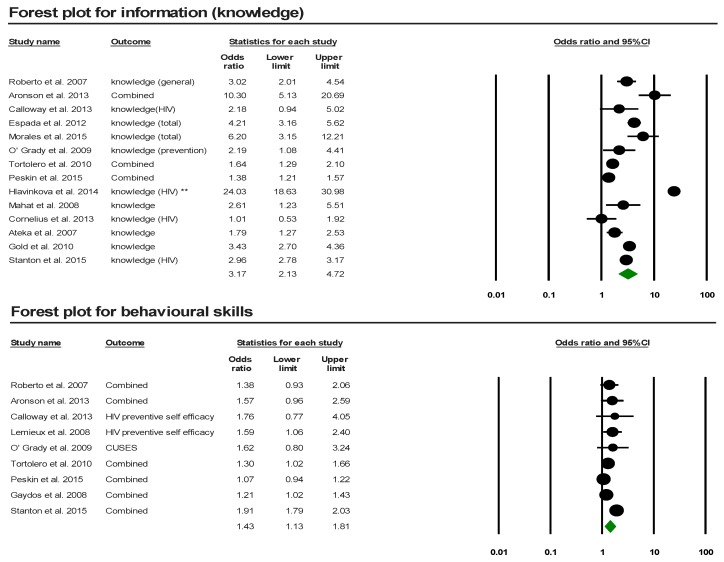
Forest plots.

Overall behavioral (OR = 1.28; 95% CI: 1.17–1.39; *I*^2^ = 0%; *p*-value = 0.65) and psychosocial (OR = 1.92; 95% CI: 1.36–2.72; *I*^2^ = 96.95%; *p*-value = 0.00) outcomes were significant compared to controls, suggesting a positive intervention effect (Table 3) (Figure 2).

#### 3.4.2. Effects of Interventions on the Psychosocial Sub-Categorical Outcomes 

The psychosocial sub-categorical variables, specifically information/knowledge (OR = 3.1; 95% CI: 2.13–4.72; *I*^2^ = 97.12%), motivation (OR = 1.69; 95% CI: 1.04–2.75; *I*^2^ = 98.67%), and behavioral skills (OR = 1.43; 95% CI: 1.13–1.81; *I*^2^ = 89.91%), were significant compared to controls, suggesting a positive intervention effect (Table 3) (Figure 2).

#### 3.4.3. Effects of Interventions on the Specific Psychosocial and Behavioral Outcomes

When examining pooled estimates of specific behavioral outcomes, sexual partners and condom use were significantly improved by the interventions. However, alcohol and/or drug use before sex and STI testing were measured by only one article and therefore prevented pooled analysis. When examining pooled estimates of specific psychosocial outcomes, attitudes (condom use and abstinence), norms and beliefs relating to condom use and abstinence, condom efficacy, HIV self-efficacy, partner communication, and parental communication were significantly improved. Information detailing specific outcomes is presented in Table 3.

#### 3.4.4. Comparative Analysis

A comparative analysis found no statistically significant differences in the effectiveness of STI interventions between the psychosocial and behavioral outcomes. Analysis at the sub-categorical level (information versus motivation + behavioral skills and information versus behavioral outcomes) found that interventions were significantly more effective for the dissemination of information compared to improving motivation and behavioral skills (*p*-value < 0.001) and behavioral outcomes (*p*-value < 0.001).

#### 3.4.5. Subgroup Analysis

There were no significant differences between subgroups based on the type of provider (peer-involved versus non-peer-involved) and type of intervention (face-to-face versus technology-based).

## 4. Discussion

Globally, youth are recognized as a vulnerable subpopulation for STIs. It is reported that more than 60% of STIs are found among this age group [14]. Our study found that students exposed to STI preventive interventions were 28% more likely to practice safe sex behaviors and 92% more likely to show improvement in psychosocial factors compared to those who were not exposed. Therefore, educational settings may represent ideal venues for the design and implementation of STI preventive intervention programs that help improve the psychosocial factors and behaviors related to disparities in sexual health among students.

From our comparative analysis, it was found that the effect of STI preventive interventions was most prominent for promoting knowledge, while some improvements were also seen for enhancing motivational factors, behavioral skills, and behavioral outcomes related to sexual practices. In the information-motivation-behavioral skills (IMB) model, behavioral change can be directly predicted by way of information (knowledge) and motivational factors and indirectly by behavioral skills [64]. If the desired behavior (i.e., carrying condoms) is not complicated and does not require developing behavioral skills, information dissemination (i.e., on the importance of carrying condoms) might have a direct impact on behavioral change [64]. However, if the desired behavior (i.e., use of a condom in every sexual encounter) has a complex nature and requires specific skills (i.e., dealing with new/casual sex partners, negotiations, self-efficacy), information dissemination alone would not be sufficient to achieve successful behavioral change [64]. Based on our findings and the concepts of the IMB model, STI preventive interventions targeting students should focus on achieving promising behavioral changes by more effectively emphasizing motivational factors and behavioral skills.

In our meta-analysis, eight of the included studies discussed the difference in the effectiveness of STI preventive interventions based on an individual’s sex. They suggest that STI preventive interventions were more effective for females compared to males [50,54,55,57,59,60,61,63] and this finding was consistent with the results reported in other research [65]. Female students showed greater improvements in knowledge and motivational factors (i.e., subjective norms, interest and confidence in safe sex behaviors, attitudes towards condom use) and were less likely to engage in risky behaviors (i.e., having multiple sexual partners) after the intervention compared to males [50,54,55,57,59,60,61,63]. These findings may be due to different social norms and expectations between sexes (sexual double standard) [66,67] and the fact that STI interventions tend to preferentially target females because they are reported to suffer more from the adverse effects of risky sexual behaviors, including unwanted pregnancies and higher rates of STIs [68].

Peer-involved programming is recognized as a key component of an effective STI prevention strategy, since youth are more likely to be influenced by their peers and aim to gain acceptance within their social group [69,70]. When examining the effectiveness of peer-involved and non-peer-involved STI interventions in our subgroup analysis, no significant difference was found. While previous systematic reviews [65,71,72] reported similar results, other studies revealed that peer-involvement had a positive impact on STI preventive interventions among youth [73,74,75]. According to Advocates for Youth, to achieve optimal results from peer-programming, adequate human and financial resources, careful and continuous recruitment, participation of peers in every step to enhance self-determination and empowerment, repeated training, and systematic supervision and evaluation of peer facilitators are required [70]. In the initial planning phase, it is important to consider multiple key factors in order to balance the cost, benefit, feasibility, and acceptability of peer-involved STI programming.

When examining the method of delivery for the different STI preventive interventions, we found that face-to-face and technology-based interventions were equally effective. Previous studies support our finding [76,77]. However, face-to-face interventions show significant effectiveness on both behavioral and psychosocial outcomes. This might be attributed to greater compliance, peer-influence, proper engagement, and sufficient dosage of delivery [78,79]. However, a recent study found that the most positive and significant outcomes were seen with the use of mixed delivery for STI interventions (i.e., combination of face-to-face and technology-based) rather than individual approaches [80]. Increasingly, youth have become reliant on the use of technology (i.e., internet, mobile phones) as part of their social environment (i.e., daily communication, information gathering, and entertainment) [81]. Therefore, given their popularity among youth, technology-based interventions have several advantages over face-to-face interventions, including broader coverage, speed, convenience, privacy, confidentiality, opportunities for open discussion, cost-effectiveness, and different delivery methods (i.e., text messaging, social networking sites, webpages, blogs, and applications) [78,79,81,82,83]. Considering the structural barriers in implementing STI preventive interventions (i.e., inadequate funding, lack of infrastructure, and limited human resources), a mixed approach (i.e., face-to-face and technology-based) may be most practical. Further research in this area is needed.

### 4.1. Strength and Limitations

This systematic review and meta-analysis has several strengths. It used a standardized, previously validated systematic methodology [84] and relied on recently published articles (last 10 years). The majority of the studies included utilized a pretest, post-test, and control group design with group-level randomization, which ensured a more accurate comparison. Additionally, our study focused specifically on educational settings in regions with similar overall STI burden, socioeconomic environments, and use of preventive strategies, which enhance comparability. Our findings provide new insights on an important research topic among a vulnerable population while also exhibiting a high level of congruence with those reported in the literature.

Despite its several strengths, our study has a few limitations. It relies on secondary data that used different statistical analyses and a variety of evaluation scales to measure the outcomes of interest, which may have led to under- or over-estimation of the pooled effect sizes. There were different post-intervention evaluation periods. To best address this issue, we used the last available evaluation period for each included study (i.e., furthest in time from the intervention). None of the included studies evaluated biological/clinical outcomes (STIs/HIV incidence and prevalence) to measure the effectiveness of their STI interventions. Finally, some of the included studies were carried out with populations that could not be entirely generalizable (i.e., small sample sizes or as pilot projects), and therefore the results of our study should be interpreted with some level of caution.

### 4.2. Recommendations for Future Research

Future research evaluating STI preventive interventions in educational settings in developed countries (including Canada, where there is scarcity of research in this area) should: (1) assess the impact of interventions by using clinical/biological outcomes to determine whether these programs contribute to the reduction of STIs; (2) evaluate the short-, intermediate-, and long-term effectiveness of the interventions by using regularly repeated follow-ups over extended periods of time; (3) examine the disparity and effectiveness of the STI interventions on the basis of differences in sex (female vs. male), type of delivery (face-to-face vs. technology-based), and type of facilitator (peer-involved vs. non-peer-involved); and (4) utilize a formative evaluation process to address the dynamic nature of the changes in the sexual behaviors of students.

## 5. Conclusions

STIs are a public health concern and pose a major burden on the health and well-being of youth. Our systematic review and meta-analysis helps to provide empirical evidence in support of the importance of comprehensive STI preventive interventions in educational settings. Such efforts are shown to have a positive impact on the students’ psychosocial factors and behaviors related to sexual health practices. To be most effective, future STI preventive interventions need to better engage male students, use a mixed delivery method (i.e., face-to-face and technology-based), and select the most appropriate type of facilitation (i.e., peer-involved and non-peer-involved). Finally, it is recommended that STI preventive interventions use a formative evaluation process to address the dynamic nature of the changes in the sexual behaviors of students and to provide them with timely supports and equitable services.

## 6. Ethics Statement

The opinions, findings, and conclusions presented/reported in this article are those of the authors and are in no way meant to represent the corporate opinions, views, or policies of the American College Health Association (ACHA). The ACHA does not warrant nor assume any liability or responsibility for the accuracy, completeness, or usefulness of any information presented in this article. This study was exempt from ethics approval because it relied on the use of ACHA micro-files and secondary analysis of anonymous data (Tri-Council Policy Statement, articles 2.2 and 2.4, respectively). Participation in this survey was voluntary.

## Figures and Tables

**Table 1:** Summary of risk of bias assessment.

Articles	Bias					Within Studies Risk of Bias
	Selection	Performance	Attrition	Detection	Reporting	
Roberto et al., 2007 [48]	+	+	+	+	+	Low risk of bias
Morales et al., 2015 [49]	+	−	+	+	+	Low risk of bias
Tortolero et al., 2010 [50]	+	−	+	+	+	Low risk of bias
Cornelius et al., 2013 [51]	+	−	+	+	+	Low risk of bias
Aronson et al., 2013 [52]	−	−	+	+	+	Moderate risk of bias
Calloway et al., 2013 [53]	−	−	+	+	+	Moderate risk of bias
Espada et al., 2012 [54]	+	−	+	+	−	Moderate risk of bias
Gaydos et al., 2008 [55]	+	−	+	−	+	Moderate risk of bias
Lemieux et al., 2008 [56]	+	−	+	−	+	Moderate risk of bias
Hlavinkova et al., 2014 [57]	−	−	+	+	+	Moderate risk of bias
Gold et al., 2010 [58]	+	−	+	−	+	Moderate risk of bias
Stanton et al., 2015 [59]	+	−	+	+	−	Moderate risk of bias
Ateka et al., 2007 [60]	+	−	−	−	+	High risk of bias
O’Grady et al., 2009 [61]	+	−	−	−	+	High risk of bias
Peskin et al., 2015 [62]	+	−	+	−	−	High risk of bias
Mahat et al., 2008 [63]	−	−	−	+	+	High risk of bias

For each bias: (+) = low risk and (−) = high risk or unclear risk. For within studies risk of bias: Low risk of bias = (+) for four or all types of bias. Moderate risk of bias = (+) for three types of bias. High risk of bias = for three or more types of bias.

**Table 2:** Summary description of the included studies.

Author, Year of Publication, and Location	Interventions		Settings	Study			
	Type	Providers		Study Design	Control	Evaluation	Characteristics of Participants
Roberto et al. [48] U.S. 2007	Computer and internet-based intervention addressing pregnancy, HIV and STIs *	No in-person provider	High school	Pretest post-test control group design (randomization at school level)	No intervention	Baseline and 10 weeks after intervention	*N* = 326 Mean age—15.5 Sex—male and female Ethnicity—European American (majority)
Morales et al. [49] Spain 2015	Culturally adapted HIV prevention and sexual health promotion program for Latino adolescents: “COMPAS (Skills for Adolescents with a Healthy Sexuality)”	Trained psychologists	High school	Pretest post-test control group design (randomization at school level)	No intervention	Baseline and 1 year after intervention	*N* = 1563 Mean age—14–16 Sex—male and female Ethnicity—Spanish
Tortolero et al. [50] U.S. 2010	Computer-based plus classroom activities for HIV, STIs, and pregnancy prevention: “IYG (Its Your Game … Keep It Real!)”	Trained facilitators	Middle school	Pretest post-test control group design (randomization at school level)	Regular health classes	Baseline and 1 year after intervention	*N* = 907 Mean age—13 Sex—male and female Ethnicity—African American
Cornelius et al. [51] U.S. 2013	Community-based HIV prevention program boosted with mobile cell phone (MCP) technology: “Becoming A Responsible Team (BART) curriculum”	Trained African American college graduate facilitators (peers)	Pilot study at university, participated by high school students	Pretest post-test treatment group only design	Baseline	Baseline, immediately, and 3 months after intervention	*N* = 40 Mean age—15.4 Sex—male and female Ethnicity—African American
Aronson et al. [52] U.S. 2013	HIV preventive intervention for black male college students: “Brothers Leading Healthy Lives”	Trained peer facilitators and educators	College	Pretest post-test treatment group only design	Baseline	Baseline, immediately, and 3 months after intervention	*N* = 54 Age range—18–24 Sex—male Ethnicity—African American
Calloway et al. [53] U.S. 2013	Preventive intervention addressing HIV and STIs for African American college students: “Playing it Safe: Protecting yourself from HIV/AIDS and other STIs”	Trained and certified peer educators	College	Pretest post-test control group design (randomization at class level)	No intervention	Baseline, immediately after intervention	*N* = 97 Mean Age—18 Sex—male and female (female 79%) Ethnicity—African American (majority)
Espada et al. [54] Spain 2012	Culturally adapted HIV prevention and sexual health promotion program for Latino adolescents: “COMPAS (Skills for Adolescents with a Healthy Sexuality)”	Trained psychologists	High school	Pretest post-test control group design (randomization at school level)	No intervention	Baseline and immediately after intervention	*N* = 827 Mean age—15.73 Sex—male and female Ethnicity—Spanish
Gaydos et al. [55] U.S. 2008	Community–university linked research and interventions addressing HIV and STIs: “Focus on Adolescents (FOA): modification of “Focus on Teens (FOT)”	Trained adult interventionists	High school	Pretest post-test treatment only group design (randomization at school level)	Baseline	Baseline, immediately, 6 months and 1 year after intervention	*N* = 1190 Mean age—14.9 Sex—male and female (female > 70%) Ethnicity—African American
Lemieux et al. [56] U.S. 2008	Music-based HIV preventive intervention	Music Opinion Leaders (MOLs) (peers)	High school	Pretest post-test control group design (randomization at school level)	Regular health classes	Baseline and 3 months after intervention	*N* = 306 Mean age—16 Sex—male and female Ethnicity—multi-ethnicity (predominantly African American and Latinos)
Hlavinkova et al. [57] Slovakia 2014	HIV prevention campaign: “Sunflower project”	Students organised, designed, and created contents of the campaign (peers)	High school and college	Pretest post-test treatment group only design	Baseline	Baseline and immediately after campaign	*N* = 533 Mean age—15.8 Sex—male and female Ethnicity—multi-ethnicity
Gold et al. [58] Australia 2010	Sexual health promotion with text messaging focusing on chlamydia screening and condom use	No in-person provider is needed; researchers, professors, and students were involved in the study	No physical setting (most participants are high school graduates)	Pretest post-test treatment group only design	Baseline	Baseline and 2 weeks after intervention	*N* = 587 Median age—22 Sex—male and female Ethnicity—not mentioned
Stanton et al. [59] Bahamas 2015	National evidence-based HIV prevention program for 6th grade students: “Focus on Youth in the Caribbean (FOYC)”	Trained teachers	Elementary school	Pretest post-test treatment group only design	Baseline	Baseline, immediately, and 1 year after intervention	*N* = 4470 (6th grade students) Mean age—10.4 Sex—male and female Ethnicity—African descendants (majority)
Ateka et al. [60] U.S. 2007	Knowledge-based adolescent sexuality program: “City of Houston HIV and STD prevention program”	Trained teachers	High school	Intervention and control comparison at post-test only	Regular health classes	Compare the data of intervention and control schools over 1 academic year	*N* = 430 Mean age—15.3 Sex—male and female Ethnicity—African American and Hispanics (Majority)
O’Grady et al. [61] U.S. 2009	Brief safe sex intervention for college students residing in residence halls: “Skills, Information, Motivation, Peer-led (SIMPL)”	Trained peer educators	College	Intervention and control comparison at post-test only	Information only	Immediately after sessions	*N* = 108 Mean age—18.85 Sex—male and female Ethnicity—White (majority)
Peskin et al. [62] U.S. 2015	Computer-based sexual health education addressing pregnancy, HIV, and STIs: “IYG tech (Its Your Game … Keep It Real!)”	Trained facilitators	Middle school	Pretest post-test control group design (randomization at school level)	No intervention	Baseline and 1 year after intervention	*N* = 1374 Mean age—14.3 Sex—male and female Ethnicity—Hispanic (74%), African American (17%), others (9%)
Mahat et al. [63] U.S. 2008	Peer Education Project (PEP) for HIV prevention: “Teens for AIDS Prevention (TAP)”	Trained peer educators (guided by nurses and teachers)	High school	Pre-test post-test control group design (no randomization)	Traditional sexual health education	Baseline and 5 months after intervention	*N* = 97 Mean age—14 Sex—male and female Ethnicity—multi-ethnicity

* HIV = Human Immunodeficiency Virus; STIs = Sexually Transmitted Infections.

**Table 3:** Summary table for pooled effect sizes of outcome measures.

Outcome Measures	# of Studies	Pooled OR	Lower Limit	Upper Limit	Heterogeneity	
					*I* ^2^	*p*-Value (Q Statistics)
**Behavioral outcomes**	10	1.28	1.17	1.39	0.00	0.648
Sexual partners	7	1.33	1.03	1.72	86.69	0.00
Sexual activity	8	1.06	0.86	1.31	64.72	0.01
Condom use	5	1.57	0.91	2.73	68.42	0.01
HIV or STI testing	1	1.26	0.93	1.72	-	-
Alcohol or drug use before sex	1	1.00	0.22	4.45	-	-
**Psychosocial outcomes**	16	1.92	1.36	2.72	96.95	0.00
Information (knowledge)	14	3.17	2.13	4.72	97.12	0.00
Motivation	13	1.69	1.04	2.75	98.67	0.00
Attitude: condom use, abstinence	6	1.37	1.10	1.69	56.63	0.04
Norms and beliefs: condom	4	1.42	1.00	2.04	70.78	0.02
Norms and beliefs: abstinence	2	1.16	1.03	1.30	0.00	0.96
Norms and beliefs: peers	4	1.07	0.96	1.19	0.00	0.70
Risk perceptions	4	2.06	0.66	6.48	98.98	0.00
Intentions (preventive behaviors)	10	1.68	0.97	2.9	99.00	0.00
Behavioral skills	9	1.43	1.13	1.81	89.91	0.00
Condom efficacy	6	1.44	1.11	1.87	89.20	0.00
Refusal self-efficacy	3	1.15	0.92	1.45	63.43	0.07
HIV self-efficacy	2	1.62	1.12	2.35	0.00	0.83
Partner communication	3	1.24	1.04	1.26	0.00	0.46
Parental communication	2	1.17	1.08	1.26	0.00	0.33
